# Frailty domains and quality of life associated with geriatric depression in Brazilian primary health care: a cross-sectional study

**DOI:** 10.1038/s41598-026-50814-6

**Published:** 2026-04-29

**Authors:** Evanilson Francisco de Moura, Bruno Araújo da Silva Dantas, Larissa Amorim Almeida, Kalyne Patrícia de Macêdo Rocha, Nathaly da Luz Andrade, Mayara Priscilla Dantas Araújo, Sandra Maria da Solidade Gomes Simões de Oliveira Torres, Maria Antónia Fernandes Caeiro Chora, Maria Laurência Grou Parreirinha Gemito, Eulália Maria Chaves Maia, Margarita del Rosario Baeza Fuentes, Carola Rosas, Sebastián Urquijo, Ana Comesaña, Gilson de Vasconcelos Torres

**Affiliations:** 1 Programa de pós-graduação em Ciências da Saúde, Universidade Federal do Rio Grande do Norte, Natal, RN Brasil; 2 Faculdade de Ciências da Saúde do Trairi, Universidade Federal do Rio Grande do Norte, Natal, RN Brazil; 3 Programa de Pós-Graduação em Saúde Coletiva, Universidade Federal do Rio Grande do Norte, Natal, RN Brazil; 4 Escola Superior de Enfermagem São João de Deus, Universidade de Évora, Évora, Portugal; 5 Departamento de Psicologia, Universidade Federal do Rio Grande do Norte, Natal, RN Brazil; 6 Universidad de Santiago de Chile, Santiago, Chile; 7 Universidad Austral de Chile, Valdivia, Chile; 8 Instituto de Investigación en Psicología Básica Aplicada y Tecnología, Universidad Nacional de Mar Del Plata, Mar del Plata, Argentina; 9 Departamento de Enfermagem, Universidade Federal do Rio Grande do Norte, Natal, RN Brazil

**Keywords:** Frailty, Quality of life, Geriatric depression, Older people, Primary health care, Diseases, Health care, Risk factors

## Abstract

Geriatric depression is a prevalent and multifactorial condition that can be influenced by domains of frailty and quality of life (QoL) in older adults. In Brazil, there is a lack of studies that explore these factors in an integrated manner within the context of Primary Health Care (PHC). Our objective was to analyze the association between frailty domains, QoL, and geriatric depression among community-dwelling older adults assisted in PHC in urban areas of low population density. A cross-sectional, quantitative study involving 323 older adults registered in PHC units from two Brazilian municipalities. The instruments used were the Edmonton Frail Scale, the Short Form Health Survey, and the Geriatric Depression Scale. Analyses included association tests, Spearman’s correlation, and univariate and multivariate binary logistic regression. There was a strong negative correlation between depression and the physical and mental health domains of QoL, particularly “mental health”. Univariate binary logistic regression indicated that poorer scores in Self-perception of health, Mood, and Functional performance dimensions of Frailty were factors independently associated with depression. However, when evaluated jointly in the multivariate analysis, Self-perception of health and Mood consistently increased the strength of association.

**Supplementary Information:**

The online version contains supplementary material available at 10.1038/s41598-026-50814-6.

## Introduction

Frailty in older adults is characterized by a decline encompassing functional impairments, mental and cognitive health deficits, and the presence of limiting chronic conditions, all of which are associated with poorer self-perceived health^1^. According to the literature, frail older individuals are more susceptible to adverse events such as falls, hospitalizations, and rapid clinical deterioration when compared to the general older population^2,3^. In the Brazilian context, the prevalence of geriatric frailty is estimated to range from 10 to 25%, reaching up to 45% among groups aged 85 years or older^4^. The interaction between this condition and Quality of Life (QoL) has been described by other authors^5^, although it remains an underexplored topic in the Latin American context.

QoL, in turn, is defined as an individual’s subjective perception of their own life, considering domains of physical and mental health, autonomy in daily activities, level of socialization, and personal values^6^. Reinforcing this perspective, Brazilian researchers have identified that QoL is strongly association by multiple factors, particularly those related to physical and mental health^7,8^.

In the field of mental health, one of the components of QoL, geriatric depression, manifests through frequent feelings of dissatisfaction with life, helplessness, hopelessness, and social withdrawal^9,10^. This is a highly relevant condition, with its prevalence in the global population estimated at 15% in a recent meta-analysis^11^. In Brazil, a prevalence of 11.8% was identified^12^, a context in which other authors have observed that depression can be both associated with and influenced by various community factors, such as advanced age, risk of abuse and violence, or functional limitations^13,14^.

Given the complexity of the interaction between frailty, QoL, and depression, Primary Health Care (PHC) plays a central role in the early identification and prevention of both chronic and acute conditions in older adults. Through comprehensive and continuous care programs, PHC promotes the monitoring of this population’s health status, enabling timely interventions that can mitigate the risk of developing conditions such as frailty and depression^15,16^.

Although other authors have analyzed frailty, QoL, and depression in different contexts^17–19^, the association of these domains with the outcome of depression has not yet been adequately measured. Studying specific dimensions of frailty and QoL implies a better understanding that geriatric depression is a multidimensional phenomenon influenced by the interaction between various domains, rather than by general concepts alone^20^. Mood changes are often the most immediate indicators of depressive symptoms^21^, while self-perception of health captures the subjective burden of clinical vulnerabilities^22^. We also prioritized delving into functional domains due to the frequent loss of autonomy in daily life, a recognized factor that triggers feelings of helplessness and social isolation, which are core components of the depressive experience in aging^13,23^.

Addressing this knowledge gap will allow for a deeper understanding of depression based on multidimensional factors, thereby supporting the development of more effective mental health policies for the prevention of this condition in geriatric and community contexts.

The objective of this study was to analyze the association between frailty domains, QoL, and geriatric depression among community-dwelling older adults assisted in PHC in urban areas of low population density. Our hypothesis was that specific domains of frailty and QoL are associated in varying degrees with depressive symptoms in this population group.

## Materials and methods

### Study design and setting

An observational, cross-sectional study with a quantitative analytical approach was conducted among older adults receiving care at PHC units in the municipalities of Santa Cruz and Macaíba, in the state of Rio Grande do Norte, Brazil. This research is part of a series of studies developed by the International Network for Research on Vulnerability, Health, Safety, and QoL of Older Adults: Brazil, Portugal, Spain, France, Chile, Mexico, and the United States of America, of which the authors are active members.

### Ethical considerations

This study was conducted in accordance with the Declaration of Helsinki on ethical research practices. It was reviewed and approved by the Research Ethics Committee of the Federal University of Rio Grande do Norte, Brazil (approval number 4.267.762), in compliance with national regulations for research involving human beings. Authorization was obtained from the local PHC authority prior to data collection. All participants provided written informed consent. For illiterate individuals, consent was obtained verbally, and fingerprint signatures were accepted. No incentives or compensation were offered for participation.

### Population and sample

The study population consisted of older adults assisted in PHC services in the participating municipalities. Using the G*Power 3.1 software, an a priori power calculation was performed to estimate the sample size required for logistic regression^24^. A statistical power of 95% was set to detect an Odds Ratio (OR) of 3.0, conservatively chosen due to the scarcity of existing evidence on associated with depression. With a significance level of 0.05, the analysis indicated a minimum sample size of 251 participants. The assumptions included a two-tailed test and an expected prevalence of 15% (Pr[Y = 1|X = 1] = 0.15), as identified in prior literature^11^. A non-probabilistic, intentional, convenience sampling method was used. A total of 323 individuals completed the study, representing a 22% increase above the initial estimate to avoid sample loss. 

Inclusion criteria were: age 60 years or older, the legal minimum in Brazil for classification as an older adult^25^; and being registered in a PHC unit in the study settings for at least six months prior to data collection. Exclusion criteria included severe cognitive impairment diagnosed by health professionals that prevented interview participation. Participants were recruited with the support of PHC professionals from the municipalities, totaling 30 units covering both urban areas. These professionals provided addresses and contact information for eligible individuals based on PHC records. This facilitated participant identification by the researchers, who actively sought them in the communities.

### Instruments and variables

Data were collected using structured questionnaires based on variables of interest. The Older Adult Health Handbook, a tool implemented in Brazilian PHC to monitor sociodemographic and health factors of older adults^26^, was used to extract data such as gender, age group, marital status, income, living alone, skin color, and educational level. Additional data included general health conditions and lifestyle habits, such as self-reported chronic diseases (Systemic Arterial Hypertension, Diabetes Mellitus, osteoporosis), polypharmacy (use of ≥ 5 medications daily), smoking, alcohol consumption, and regular physical exercise or socialization.

Frailty was assessed using the Edmonton Frail Scale (EFS), which covers nine domains: cognition, previous hospitalization, self-perception of health, emotional independence, social support, polypharmacy, medication memory, nutrition, mood, continence, and functional performance. The total score ranges from 0 to 17 points, classifying individuals as “Not frail (0–4),” “Apparently vulnerable (5–6),” “Mild frailty (7–8),” “Moderate frailty (9–10),” and “Severe frailty (> 11)”^1^. For this analysis, both the total score and domain-specific scores were used as continuous independent variables. Internal reliability was α = 0.71 (substantial).

QoL was measured using the Medical Outcomes Study Short Form-36 (SF-36), which includes 36 items across eight domains: physical role functioning, physical functioning, pain, general health perceptions, vitality, social role functioning, emotional role functioning, and mental health, grouped into two dimensions: physical health and mental health. Domain and dimension scores were calculated on a 0–100 scale^27^ and used as independent variables in the analyses. Instrument reliability was α = 0.84 (almost perfect).

Depression was measured using the 15-item version of the Geriatric Depression Scale (GDS-15). This scale consists of dichotomous (“yes” or “no”) questions, formulated in a simple and direct way to facilitate comprehension by older adults. Items address emotional, cognitive, and social domains of depressive mood, such as life satisfaction, negative feelings, and perceived energy for routine activities. The total score ranges from 0 to 15 points, with each depression-indicative answer scoring 1 point and non-indicative answers scoring 0. Final classification is: no relevant depressive symptoms (0–4), mild depression (5–8), moderate depression (9–11), and severe depression (12–15)^9,10^. For the present study, depression was recategorized as “No depression” (0–4) and “With depression” (5–15), forming the comparison groups and primary outcome categories. The total score was also used as a scalar variable for analyses. Internal reliability in this study was α = 0.72 (substantial).

All instruments were adapted and validated for Brazilian Portuguese.

### Data collection

Data collection occurred by convenience of the researchers, through face-to-face interviews conducted in Brazilian Portuguese, using structured paper instruments. Participants selected the options that best represented their responses, which were recorded by the interviewer. Data collection occurred between June 2023 and March 2024, conducted by 16 university students from various health fields. Observer variation was minimized through a structured training methodology. All students were trained to conduct full interviews using all instruments for each participant. Training was conducted in three stages by the coordinator: (1) theoretical foundation through classroom slide presentations; (2) simulation of data collection in which each student conducted at least two pilot interviews with individuals not included in the study sample; (3) point-by-point discussion of item interpretation to ensure consistency among researchers. Each interview lasted approximately 60 min and was conducted at participants’ homes or at university facilities, depending on participant or researcher convenience. No blinding was implemented, and no randomization was applied for group allocation.

### Data analysis

Data were analyzed using the Statistical Package for the Social Sciences (SPSS), version 23.0. Descriptive analyses were performed using absolute and relative frequencies for categorical variables. Kolmogorov-Smirnov tests indicated non-normal distribution. Pearson’s chi-square test was applied to assess associations between categorical variables, and odds ratios (OR) were calculated to estimate potential confounding factors between sociodemographic and health variables (OR > 1.00 with 95% confidence interval). For variables with missing data, the proportions of each category were weighted according to the study group size in which the loss occurred, so that p-values and ORs reflected representative percentages.

Spearman’s correlation was used to evaluate the strength between the total GDS-15 score and independent variables. Strength was classified as: *r* < 0.29 (weak); 0.30 ≤ *r* ≤ 0.49 (moderate); *r* ≥ 0.50 (strong). Univariate binary logistic regression was implemented to evaluate the association between individual domains of frailty and QoL with geriatric depression. To be included in the subsequent multivariate stage, variables had to meet the requirements of tolerance > 0.1 and Variance Inflation Factor (VIF) < 10 to avoid multicollinearity. Notably, the EFS Total Score was assessed in the univariate analysis but excluded from the multivariate model due to multicollinearity with its specific domains, which were prioritized to identify distinct therapeutic targets. For the final analysis, a single, complete multivariate binary logistic regression model (Enter method) was performed, including all significant factors and potential confounding factors (sociodemographic and health variables) simultaneously. The Nagelkerke R^2^ was used to assess the overall model fit. The analysis prioritized individual EFS domains to identify specific clinical targets, as aggregate scores may mask distinct associations between psychological, functional, and social dimensions of frailty and geriatric depression. Statistical significance was set at *p* < 0.05 with a 95% confidence interval^28^.

Cronbach’s alpha was used to assess the internal reliability of instruments, with the following thresholds: α = 0–0.21 (slight); α = 0.21–0.40 (fair); α = 0.41–0.60 (moderate); α = 0.61–0.80 (substantial); α = 0.81–1.00 (almost perfect)^29^.

## Results

The initial sample consisted of 323 individuals, of whom *n* = 244 (75.5%) did not have depression. The mean age was 73.1 years (SD = 8.9), and no participants were excluded from the analysis.

Participants were predominantly women (*n* = 217/ 67.2%), aged between 60 and 79 years (*n* = 247/ 76.5%), married or cohabitating (*n* = 172/ 53.3%), with income above the minimum wage (*n* = 209/ 64.7%), not living alone (*n* = 269/ 83.3%), multiracial (including Black, mixed-race, and Indigenous) (*n* = 192/ 59.4%), and literate (*n* = 249/ 77.1%). Comparative analysis between the study groups did not reveal any significant associations, suggesting similarity between them.

Table 1 presents the participants’ health and lifestyle profiles. A higher prevalence of chronic diseases was found in the depression group. The habits of engaging in regular physical activity and maintaining social interaction with others behaved as important protective factors against the presence of depression.

**Table 1:** Association analysis between depression, health profile, and lifestyle habits (*n* = 323).

Health profile and lifestyle habits		Symptoms of geriatric depression- GDS-15							
		No depression (*n* = 244)		With depression (*n* = 79)		Total (*n* = 323)		*p* ^a^	OR (CI 95%)^b^
		n	%	n	%	n	%		
Self-reported chronic diseases	Yes	192	78.7	72	91.1	264	81.7	0.013	1.21 (1.07–1.36)^c^
	No	52	21.3	7	8.9	59	18.3		
Polypharmacy	Yes	34	13.9	18	22.8	52	16.1	0.063	0.65 (0.42–1.00)
	No	210	86.1	61	77.2	271	83.9		
Smoking^d^	Yes	27	11.4	9	11.5	36	11.4	0.972	1.01 (0.55–1.84)
	No	210	88.6	69	88.5	279	88.6		
Alcoholism^d^	Yes	22	9.3	8	10.3	30	9.5	0.799	1.09 (0.58–2.03)
	No	215	90.7	70	89.7	285	90.5		
Regular physical activity^e^	Yes	122	51.7	23	29.5	145	46.2	0.001	2.05 (1.33–3.16)^c^
	No	114	48.3	55	70.5	169	53.8		
Social interaction regularly^e^	Yes	155	65.4	29	37.7	184	58.6	< 0.001	2.34 (1.57–3.50)^c^
	No	82	34.6	48	62.3	130	41.4		

OR, odds ratio; CI 95%, confidence interval 95%; GDS-15, geriatric depression scale.

^a^Pearson’s Chi-Square Test.

^b^OR for cohort “With depression”.

^c^OR for cohort “No depression”.

^d^ 8 Missing data.

^e^9 Missing data.

Table 2 describes the associations between frailty and QoL scalar variables according to the presence of depression. Regarding frailty, we found associations with Self-perception of health, Functional independence, Social support, Memory for medicines, Mood, Continence, and Functional performance. For QoL, all domains and dimensions behaved similarly.

**Table 2:** Association between continuous variables of frailty and quality of life with geriatric depression (*n* = 323).

Multidimensional domains	Symptoms of geriatric depression- GDS-15				
	No depression (*n* = 244)		With depression (*n* = 79)		*p* ^a^
	Percentile 25–50–75	Mean (SD)	Percentile 25–50–75	Mean (SD)	
Frailty (EFS)					
Knowledge	0.0–1.0–2.0	1.1 (0.9)	0.0–2.0–2.0	1.2 (0.8)	0.159
Previous hospitalization	0.0–0.0–0.0	0.1 (0.3)	0.0–0.0–0.0	0.2 (0.5)	0.090
Self-perception of health	0.0–0.0–1.0	0.4 (0.5)	0.0–1.0–2.0	0.9 (0.7)	< 0.001
Functional independence	0.0–0.0–1.0	0.4 (0.7)	0.0–1.0–2.0	0.9 (0.8)	< 0.001
Social support	0.0–0.0–0.0	0.1 (0.4)	0.0–0.0–0.0	0.3 (0.5)	0.021
Polypharmacy	0.0–0.0–0.0	0.2 (0.3)	0.0–0.0–1.0	0.3 (0.4)	0.059
Memory for medicines	0.0–0.0–1.0	0.3 (0.5)	0.0–1.0–1.0	0.6 (0.5)	0.001
Nutrition	0.0–0.0–0.0	0.2 (0.4)	0.0–0.0–0.0	0.2 (0.4)	0.679
Mood	0.0–0.0–1.0	0.3 (0.4)	1.0–1.0–1.0	0.8 (0.4)	0.001
Continence	0.0–0.0–1.0	0.3 (0.5)	0.0–0.0–1.0	0.4 (0.5)	0.028
Functional performance	0.0–1.0–1.0	0.7 (0.6)	0.0–1.0–2.0	1.1 (0.7)	< 0.001
Total Score	2.0–4.0–6.0	4.2 (2.6)	4.0–7.0–10.0	6.9 (3.2)	< 0.001
QoL - SF-36 domains					
Physical role functioning	50.0–80.0–95.0	69.1 (28.4)	10.0–45.0–75.0	43.4 (34.2)	< 0.001
Physical functioning	25.0–100.0–100.0	70.7 (41.0)	0.0–0.0–100.0	36.0 (44.7)	< 0.001
Pain	51.0–72.0–90.0	66.2 (22.6)	31.0–50.0–62.0	50.4 (24.8)	< 0.001
General health perceptions	35.0–45.0–55.0	43.3 (14.7)	20.0–30.0–45.0	30.4 (17.1)	< 0.001
Vitality	60.0–70.0–80.0	69.4 (14.8)	40.0–55.0–65.0	52.5 (16.0)	< 0.001
Social role functioning	88.0–100.0–100.0	88.2 (16.6)	50.0–75.0–88.0	69.2 (24.2)	< 0.001
Emotional role functioning	56.0–72.0–84.0	68.4 (18.1)	44.0–52.0–68.0	54.1 (16.8)	< 0.001
Mental health	64.0–72.0–84.0	71.8 (13.4)	40.0–52.0–68.0	52.3 (16.9)	< 0.001
Total score	59.0–70.5–79.0	68.4 (13.5)	37.0–46.0–62.0	48.5 (16.3)	< 0.001
Dimensions					
Physical health	51.3–67.1–78.4	63.7 (17.1)	28.2–37.0–57.2	42.5 (19.4)	< 0.001
Mental health	61.0–69.0–77.0	68.2 (10.5)	40.0–52.0–63.0	51.7 (13.5)	< 0.001

GDS-15, geriatric depression scale; SD, standard deviation; EFS, Edmonton frailty scale; QoL, quality of life; SF-36, medical outcomes study questionnaire short form.

^a^Mann–Whitney test.

Table 3 shows the correlation analysis between depression scores (GDS-15) and independent variables. A moderate correlation was found with Self-perception of health and Memory for medicines, and a strong correlation with Mood of frailty. The direction of the scales indicated that the frailer the individuals, the higher their levels of depression.

**Table 3:** Correlation analysis between depression score (GDS-15) and domains of frailty (EFS) and QoL (SF-36) (*n* = 323).

Multidimensional domains	Geriatric depression (GDS-15)	
	*r* ^a^	*p* ^b^
Frailty (EFS)		
Knowledge	0.17	0.037
Previous hospitalization	0.08	0.147
Self-perception of health	0.36	< 0.001
Functional independence	0.28	< 0.001
Social support	0.08	0.136
Polypharmacy	0.24	< 0.001
Memory for medicines	0.32	< 0.001
Nutrition	0.04	0.504
Mood	0.51	< 0.001
Continence	0.16	0.003
Functional performance	0.29	< 0.001
Total Score	0.46	< 0.001
QoL - SF-36 domains		
Physical role functioning	− 0.42	< 0.001
Physical functioning	− 0.35	< 0.001
Pain	− 0.37	< 0.001
General health perceptions	− 0.42	< 0.001
Vitality	− 0.47	< 0.001
Social role functioning	− 0.38	< 0.001
Emotional role functioning	− 0.37	< 0.001
Mental health	− 0.53	< 0.001
Total score	− 0.56	< 0.001
Dimensions		
Physical health	− 0.53	< 0.001
Mental health	− 0.55	< 0.001

Correlation levels: *r* < 0.29 (weak); 0.29 *≥* *r* *≤* 0.49 (moderate); *r* *≥* 0.50 (strong).

GDS-15, geriatric depression scale; EFS, Edmonton frailty scale; QoL, quality of life; SF-36, medical outcomes study questionnaire short form.

^a^Correlation coefficient.

^b^Spearman’s correlation test.

Regarding QoL, all domains showed moderate correlations, except for Mental health, which showed a strong correlation. Similarly, the combined domains representing the Physical health and Mental health dimensions exhibited strong correlation coefficients.

Finally, we implemented a binary logistic regression model for independent variables eligible under the established requirements (Table 4). This analysis aimed to measure their strength of association with the outcome (With symptoms). Only some frailty variables were included, among which Self-perception of health, Mood, and Functional performance stood out.

**Table 4:** Univariate and multivariate binary logistic regression between the presence of depression and independent variables (*n* = 323).

Multidimensional domains	Binary Logistic Regression				
	*R* ^2^ ^a^	*p* ^b^	ß (S.E)^c^	Wald	OR (CI 95%)
Univariate analysis					
Frailty (EFS)					
Knowledge	0.01	0.162	0.21 (0.15)	1.95	1.23 (0.92–1.64)
Previous hospitalization^d^	0.01	0.068	0.53 (0.29)	3.33	1.71 (0.96–3.03)
Self-perception of health	0.18	< 0.001	1.29 (0.21)	36.58	3.62 (2.39–5.50)
Functional Independence	0.10	< 0.001	0.79 (0.17)	21.82	2.19 (1.58–3.05)
Social support	0.02	0.023	0.59 (0.26)	5.13	1.80 (1.08–3.00)
Polypharmacy	0.02	0.044	0.58 (0.29)	4.07	1.79 (1.02–3.14)
Memory for medicines	0.05	0.002	0.77 (0.24)	10.03	2.16 (1.34–3.48)
Nutrition	0.00	0.728	0.10 (0.30)	0.12	1.11 (0.62–2.00)
Mood	0.24	< 0.001	2.11 (0.30)	48.54	8.26 (4.56–14.95)
Continence	0.02	0.035	0.55 (0.26)	4.45	1.74 (1.04–2.91)
Functional performance	0.08	< 0.001	0.84 (0.20)	17.63	2.31 (1.56–3.42)
Total score	0.21	< 0.001	0.33 (0.05)	41.81	1.38 (1.25–1.53)
Multivariate analysis - Forward Stepwise Model					
Frailty (EFS)					
Block 1					
Self-perception of health	0.18	< 0.001	1.29 (0.21)	36.58	3.62 (2.39–5.50)
Block 2					
Self-perception of health	0.32	< 0.001	1.02 (0.23)	19.97	2.77 (1.77–4.33)
Mood		< 0.001	1.85 (0.31)	34.48	6.34 (3.42–11.74)

Variables from the EFS (Knowledge, Previous hospitalization, Functional independence, and Nutrition) and SF-36 domains were excluded from the multivariate models based on two criteria: (1) lack of statistical significance in the univariate analysis (*p* > 0.05); or (2) presence of multicollinearity, as indicated by a Variance Inflation Factor (VIF) > 10. The EFS Total Score was analyzed individually in the univariate stage but excluded from the final adjusted model to avoid redundancy with its specific domains.

OR, odds ratio; CI 95%, confidence interval 95%, EFS, Edmonton frailty scale.

^a^R^2^ de Nagelkerke (Model fit).

^b^Model (Forward LR).

^c^Unstandardized coefficient.

^d^The variable was not eligible for the regression model and, therefore, was not calculated.

These variables were then entered into a single multivariate binary logistic regression model (Enter method) to identify factors independently associated with geriatric depression while simultaneously controlling for potential confounding factors (gender, age range, living alone, needing help leaving home, skin color, chronic diseases, physical activity, and social interaction). The final adjusted model showed a robust fit, with a Nagelkerke R2 coefficient of 0.39. Within this complete model, Self-perception of health (adjusted OR: 2.85; 95% CI 1.45–5.57) and Mood (adjusted OR: 5.53; 95% CI 2.64–11.60) remained the only factors significantly associated with the presence of depressive symptoms. This indicates that these domains are robust factors associated with the outcome, maintaining their significance even after full adjustment for sociodemographic and health-related variables.

The lack of statistical significance for sociodemographic and lifestyle variables in the final model reinforces that the associations found are independent of the participants’ social and health profiles. This demonstrates that these frailty domains are robust indicators of depression regardless of the elderly person’s gender, age, or socioeconomic context. Detailed results are presented in Table S1.

## Discussion

The main findings of this study highlighted the association between specific domains of frailty, QoL, and the presence of depression in older adults receiving PHC. The domains related to memory, general health perception, functionality, physical health, emotional aspects, and mental health stood out. More refined binary logistic regression analyses revealed self-perception of health and frailty-related mood as the main factors independently associated with depression, consistently increasing when jointly assessed in the multivariate analysis. QoL was not eligible for logistic regression. We believe these results provide a relevant comparative parameter with high replicability in similar settings by investigating, in an integrated and analytical way, the relationship between these constructs and depression within the PHC context, a strategic setting for mental health surveillance and promotion in aging. However, individual particularities such as sociodemographic and health profiles must be considered.

Regarding the sociodemographic profile of the sample, our findings are consistent with previous studies, such as the predominance of women, non-White individuals, and literate participants^30–32^. A study involving Brazilian and Portuguese older adults found that female gender, low income, and low education were determining factors for low QoL among depressive participants^30^. In this context, we considered gender, age range, living alone, need for help leaving home, and skin color for adjusted analysis. However, no sociodemographic variable appeared to be meaningfully significant association with depression and frailty.

As for the health profile of our sample, participants had a high prevalence of chronic diseases. However, intergroup comparison showed that individuals without depression had healthier habits, particularly regular physical exercise and socialization, which emerged as important protective factors against depression in our results. These findings suggest that such habits are beneficial in reducing depressive symptoms, as also evidenced by previous studies reporting improvements in geriatric depression with interventions focusing on physical fitness and social activities^33^. A systematic review of 15 randomized clinical trials identified significant reductions in depression levels with multidimensional and mind-body exercise programs compared with unimodal exercises^34^. Therefore, we also included physical and social activity habits in the adjusted binary logistic regression analysis, which did not indicate them as consistent confounding factors.

When delving into frailty domains, intergroup association analysis showed important differences in scores across several domains. However, those with strong correlations and associations in more robust analyses were Self-perception of health, Functional performance, and Mood, with the latter indicating that individuals with impairments had up to eight times greater odds of developing depression in univariate binary logistic regression. In the multivariate model, we observed a consistent increase in the strength of association for Mood when included alongside Self-perception of health, suggesting that the way individuals perceive their overall health is linked to their mood. The high adjusted OR for Mood and Self-perception of health are consistent with the study’s scoring system. In the Edmonton Frail Scale, higher scores reflect worse health or frailty. Since the outcome was the presence of depressive symptoms, an adjusted OR correctly indicates that poorer mood or health perception increases the likelihood of depression. This mathematical consistency confirms these domains as robust factors associated with the outcome.

The strong correlation between the SF-36 Mental Health domain and GDS-15 was expected, as both instruments capture facets of psychological well-being. However, to avoid circular reasoning in our adjusted models, we prioritized frailty domains that offer independent clinical insights into the older adult’s vulnerability.

Although the association of mood-related domains is predictable, it should be noted that functional domains also emerged as factors associated with depression, albeit with smaller proportions. The literature explains that functional dependence, particularly regarding limitations in basic and instrumental activities of daily living, imposes a series of psychological consequences on older adults, amplifying frustrations and even influencing self-perception of physical health^35^. In this context, Chinese authors found that depression is an important mediator of both functional ability decline and cognitive performance^23,36^. Frailty-related functional impairments have also been linked to various adverse outcomes with high morbidity and mortality potential, such as falls^3,5,37^.

The findings from our model adjusted for sociodemographic and health variables represent robust evidence of this study, demonstrating that Mood and Self-perception of health are independently associated with geriatric depression. Unlike previous studies that often emphasize sociodemographic determinants such as gender or age in isolation^38–40^, our analysis shows that these specific frailty domains maintain a strong association with the outcome even when adjusted for a wide range of potential confounding factors, including chronic diseases and lifestyle habits. Specifically, the high adjusted Odds Ratio for Mood suggests that this domain is a primary indicator of depressive symptoms in the Brazilian PHC context, regardless of the participants’ social profile.

It is worth noting that the EFS instrument used in our study evaluates functionality in two ways: (1) by asking participants about their abilities to perform instrumental activities of daily living, such as meal preparation, independent mobility, and household chores; and (2) through direct observation of functional performance during tasks such as standing up and walking a specified distance^1^. Although Functional performance proved important in the univariate analysis, its association was not confirmed when included in the multivariate model.

Although not eligible for logistic regression analysis, QoL variables exhibited strong correlations in both physical and mental health dimensions. Among the domains assessed, Mental health was the most prominent. Vitality and general health perception also stood out as subjective perceptions of QoL, since the SF-36 evaluates them based on participants’ feelings about their own health and their energy or disposition to engage in vigorous or non-vigorous activities^27^.

Functional and physical domains also emerged within QoL domains, while pain and social functioning were relevantly correlated with depression. Different studies have identified pain as a prevalent symptom in older adults, commonly associated with chronic diseases and conditions, including depression^41,42^. Other authors have pointed to its limiting and disabling potential for numerous activities and movements, including social interactions, particularly when mobility is required^43^. This relationship with social interaction is also highlighted in the literature as a factor influencing depression and vice versa^44,45^. This aligns with our findings, given that our sample without depression regularly engaged in physical and social activities.

Overall, this study provides a detailed diagnostic analysis of depression and its main associated factors (frailty and QoL) in a population profile common in developing countries. Thus, our findings may support consistent pathways for designing mental health prevention and protection policies for older adults within PHC. Screening and diagnosis of depression and other mental health disorders are recommended in the Comprehensive Mental Health Action Plan 2013–2030, developed by the World Health Organization^46^.

In Brazil, researchers found good acceptability for collaborative and multiprofessional care interventions involving behavioral activation through a smartphone app with educational video resources^47^. Moreover, the use of group messaging apps proved to be common even among low-income older adults, suggesting an important avenue for expanding telehealth actions against depressive disorders and improving QoL^48^.

In the international context, Chinese researchers observed significant improvements in depressive symptoms and cognition with the implementation of music therapy in older adults with cognitive deficits^49^. A systematic review found moderate evidence of effectiveness with animal-assisted therapy using trained companion animals for older adults, reducing depression levels in intervention groups^50^.

Continuous professional training, integration with specialized mental health care, and longitudinal follow-up should also be considered at the governmental level. For instance, the Improving Mood – Promoting Access to Collaborative Treatment model implemented in the United States is a successful strategy based on collaborative management involving physicians, psychologists, social workers, and case managers who systematically monitor patients through individualized care plans and screening tools^51^. This model has already demonstrated substantial improvements in depression levels among the populations served^52,53^.

These interventions are directly related to the findings of the present study, particularly regarding the need to address psychosocial domains most implicated in depression. Recognizing that domains such as mood, health perception, and vitality are effective therapeutic targets reinforces the urgency of strengthening psychosocial, educational, and cultural actions within PHC territories.

Nevertheless, the external validity of our results should be interpreted with caution, as we obtained a non-probabilistic sample restricted to a specific region of Brazil, allowing comparison only with other medium-sized urban contexts with similar socioeconomic characteristics.

### Limitations

This study has limitations. The cross-sectional design does not allow for causal inference between frailty domains, QoL, and depression symptoms. Another limitation is the use of non-probabilistic, convenience sampling, which may introduce selection bias and compromise the external validity of findings. The study may also be subject to information bias due to the use of self-reported instruments, as responses may have been influenced by momentary factors such as emotional state or interview context.

## Conclusion

We found that self-perception of health and mood, as frailty domains, were the factors most strongly associated with geriatric depression in our sample. Although QoL was not eligible for more refined analyses, both physical and mental aspects emerged as relevant associations with higher levels of depression. These results support our study hypothesis.

## Supplementary Information

Legend for table S1: R^2^ de Nagelkerke (Model fit): 0.39; ^a^ Unstandardized coefficient;^b^ Model (Enter); Abbreviations: OR: Odds Ratio; CI 95%: Confidence Interval 95%; EFS: Edmonton Frailty Scale. Note: Results were obtained through a single, complete Multivariate Binary Logistic Regression model (Enter method). The dependent variable is the presence of geriatric depressive symptoms (GDS-15 ≥ 5). Nagelkerke R2 = 0.39 indicates the overall model fit, showing the proportion of variance explained by the set of variables included. Adjusted Odds Ratios (aOR) represent the association of each variable while simultaneously controlling for all other predictors and confounding factors in the model. Self-perception of health (aOR: 2.85; 95% CI: 1.45–5.57) and Mood (aOR: 5.53; 95% CI: 2.64–11.60) remained the only factors robustly associated with the outcome after full adjustment (p<0.05).                                                                                                                                                                                                                                                                                                                                                                                                                                                      Below is the link to the electronic supplementary material.

Supplementary Material 1

## Data Availability

The data that support the findings of this study are openly available in Figshare at 10.6084/m9.figshare.30347158.v1.
