# Supplementary material for: Frailty domains and quality of life associated with geriatric depression in Brazilian primary health care: a cross-sectional study
Source: Sci Rep. 2026 Apr 29;16:19846. doi: 10.1038/s41598-026-50814-6 (PMC13315697; doi:10.1038/s41598-026-50814-6)
Supplement: Supplementary file 1 — Supplementary Material 1 [file 41598_2026_50814_MOESM1_ESM.docx]

**Table S1.** Multivariate binary logistic regression analysis of factors associated with geriatric depression (n=323).

| **Variables** | **Binary Logistic Regression - Adjusted** | | | |
| --- | --- | --- | --- | --- |
|  | **ß (S.E)** ^a^ | **Wald** | **p** ^b^ | **OR (CI 95%)** |
| **Main Frailty Domains (EFS)** |  |  |  |  |
| Self-perception of health | 1.05 (0.34) | 9.33 | 0.002 | 2.85 (1.45 - 5.57) |
| Social support | 0.55 (0.39) | 2.00 | 0.157 | 1.73 (0.81 - 3.72) |
| Polypharmacy | 0.02 (0.38) | 0.01 | 0.956 | 1.02 (0.49 - 2.14) |
| Memory for medicines | 0.54 (0.35) | 2.35 | 0.125 | 1.72 (0.86 - 3.45) |
| Mood | 1.71 (0.38) | 20.51 | <0.001 | 5.53 (2.64 - 11.60) |
| Continence | -0.12 (0.36) | 0.11 | 0.740 | 0.89 (0.44 - 1.80) |
| Functional performance | -0.16 (0.35) | 0.21 | 0.646 | 0.85 (0.42 - 1.70) |
| Total Score | -0.01 (0.13) | 0.01 | 0.982 | 1.00(0.77 - 1.28) |
| **Confounding Factors** |  |  |  |  |
| Gender (Woman) | -0.40 (0.36) | 1.20 | 0.273 | 0.67 (0.33 - 1.37) |
| Age range, yr (> 80) | -0.01 (0.43) | 0.0 | 0.991 | 0.99 (0.43 - 2.32) |
| Live alone (Yes) | -0.02 (0.46) | 0.01 | 0.972 | 0.98 (0.40 - 2.44) |
| Need help leaving home (Yes) | -0.28 (0.41) | 0.47 | 0.495 | 0.76 (0.34 - 1.69) |
| Skin color (No white) | -0.14 (0.35) | 0.17 | 0.678 | 0.87 (0.44 - 1.71) |
| Self-reported chronic diseases (Yes) | 0.44 (0.53) | 0.70 | 0.402 | 1.56 (0.55 - 4.37) |
| Regular physical activity (No) | 0.54 (0.37) | 2.10 | 0.150 | 1.71 (0.82 - 3.55) |
| Social interaction regularly (No) | 0.63 (0.37) | 2.89 | 0.089 | 1.88 (0.91 - 3.90) |

R^2^ de Nagelkerke (Model fit): 0.39; ^a^ Unstandardized coefficient; ^b^ Model (Enter); Abbreviations: OR: Odds Ratio; CI 95%: Confidence Interval 95%; EFS: Edmonton Frailty Scale. Note: Results were obtained through a single, complete Multivariate Binary Logistic Regression model (Enter method). The dependent variable is the presence of geriatric depressive symptoms (GDS-15 ≥ 5). Nagelkerke R2 = 0.39 indicates the overall model fit, showing the proportion of variance explained by the set of variables included. Adjusted Odds Ratios (aOR) represent the association of each variable while simultaneously controlling for all other predictors and confounding factors in the model. Self-perception of health (aOR: 2.85; 95% CI: 1.45–5.57) and Mood (aOR: 5.53; 95% CI: 2.64–11.60) remained the only factors robustly associated with the outcome after full adjustment (p<0.05).
